# Nine Years of Chronic Myeloid Leukemia in a Young Adult With a Variant Philadelphia Chromosome: A Case Report and Brief Literature Review

**DOI:** 10.7759/cureus.113311

**Published:** 2026-07-24

**Authors:** Alireza Izadian Bidgoli, Alberto Gomez Usatorres, Alberto Gomez Veliz, Angela C Gallagher, Daine Noriega-Toledo

**Affiliations:** 1 Internal Medicine, American University of the Caribbean School of Medicine, Cupecoy, SXM; 2 Internal Medicine, Jackson Memorial Hospital, Miami, USA; 3 Internal Medicine, Universidad Hispanoamericana, San Jose, CRI; 4 Internal Medicine, American University of the Caribbean, Miami, USA; 5 Flow Cytometry Department, Jackson Memorial Hospital, Miami, USA

**Keywords:** bcr::abl1, chronic myeloid leukemia (cml), splenomegaly, variant philadelphia chromosome, young adult

## Abstract

We report the case of a man diagnosed with chronic-phase chronic myeloid leukemia (CML) at 24 years of age after marked leukocytosis was incidentally identified during routine blood donation screening. Initial evaluation demonstrated a white blood cell count of 187.8 ×10³/µL, anemia, basophilia, and left-shifted myeloid proliferation. Bone marrow examination confirmed chronic-phase CML, and cytogenetic analysis identified a rare variant Philadelphia chromosome translocation, t(6;9;22)(q34;q11.2;p21). Fluorescence in situ hybridization confirmed BCR::ABL1 rearrangement in 94.5% of analyzed cells.

Nine years after diagnosis, the patient was hospitalized for evaluation of persistent disease. Repeat bone marrow examination demonstrated markedly hypercellular marrow (95% cellularity) with myeloid hyperplasia, decreased erythropoiesis, and moderate reticulin fibrosis (MF-2), without evidence of accelerated-phase or blast-phase transformation. Peripheral blood flow cytometry identified a small CD34-positive immature myeloid population (3.8-4.3% of leukocytes) without immunophenotypic evidence of acute leukemic transformation. Computed tomography revealed massive splenomegaly measuring 31.9 cm with associated mass effect on adjacent abdominal structures.

This case illustrates the long-term clinical course of chronic-phase CML in a young adult with a rare variant Philadelphia chromosome translocation. Despite persistent hematologic abnormalities, marked splenomegaly, and moderate marrow fibrosis nearly a decade after diagnosis, the patient remained in chronic phase without leukemic transformation. The case highlights the importance of comprehensive cytogenetic evaluation, accurate disease-phase assessment, and long-term molecular surveillance in patients with uncommon cytogenetic variants.

## Introduction

Chronic myeloid leukemia (CML) is a clonal myeloproliferative neoplasm driven by the BCR::ABL1 fusion gene, which results from the Philadelphia chromosome translocation and leads to constitutive tyrosine kinase activation [1-3]. The introduction of tyrosine kinase inhibitors (TKIs) has transformed CML from a fatal malignancy into a chronic disease with excellent long-term outcomes for many patients [1-3].

CML is predominantly diagnosed in older adults, with a median age at diagnosis of approximately 60-65 years [1-3]. In contrast, adolescents and young adults represent a relatively small proportion of newly diagnosed cases and often present with higher leukocyte counts, larger spleen size, and greater disease burden at diagnosis [4-7]. As survival continues to improve, understanding the long-term clinical course of CML in younger patients has become increasingly important [4,6,7].

Although most patients harbor the classic t(9;22)(q34;q11.2) Philadelphia chromosome, variant Philadelphia chromosome translocations involving additional chromosomes occur in approximately 5-10% of cases [1,8-11]. The prognostic significance of these uncommon rearrangements remains incompletely understood, particularly for rare three-way translocations involving chromosome 6 [8-14].

We report a case of BCR::ABL1-positive chronic-phase CML diagnosed at 24 years of age in a patient harboring a rare variant Philadelphia chromosome t(6;9;22)(q34;q11.2;p21). Nine years after diagnosis, he remained in chronic phase despite persistent disease burden characterized by marked leukocytosis, massive splenomegaly, marrow fibrosis, and persistent BCR::ABL1 positivity.

## Case presentation

Clinical presentation

A 33-year-old man with a history of BCR::ABL1-positive chronic-phase chronic myeloid leukemia (CML) presented for evaluation of persistent disease approximately nine years after his initial diagnosis. He was first diagnosed with CML in 2017 at 24 years of age after marked leukocytosis was incidentally identified during routine blood donation screening. Initial laboratory studies revealed a white blood cell count of 187.8 ×10³/µL, hemoglobin of 10.3 g/dL, and platelet count of 418 ×10³/µL. Peripheral blood smear demonstrated marked leukocytosis with left-shifted myeloid maturation, basophilia, and approximately 2% circulating blasts. Bone marrow evaluation showed a hypercellular marrow with granulocytic hyperplasia and left-shifted myeloid maturation consistent with chronic-phase CML. Cytogenetic analysis identified a variant Philadelphia chromosome translocation, t(6;9;22)(q34;q11.2;p21), and fluorescence in situ hybridization (FISH) confirmed BCR::ABL1 fusion positivity in 94.5% of analyzed cells, establishing the diagnosis of BCR::ABL1-positive chronic-phase CML. Table 1 summarizes the timeline from 2017 until current presentation. 

**Table 1:** Timeline of the patient's clinical course, treatment, and disease assessment Chronic-phase CML diagnosed at age 24 with a variant Philadelphia chromosome (t(6;9;22)); multiple treatment interruptions due to insurance and loss to follow-up; persistent chronic-phase disease over nine years without progression to accelerated or blast phase despite hyperleukocytosis, marrow fibrosis (MF-2), and massive splenomegaly. BCR::ABL1 - breakpoint cluster region-Abelson 1; CBC - complete blood count; CML - chronic myeloid leukemia; CT - computed tomography; FISH - fluorescence in situ hybridization; MF - myelofibrosis grade; TKI - tyrosine kinase inhibitor; WBC - white blood cell count

Date	Clinical course	Key findings	Treatment	Response/outcome
Apr 2017	Incidental leukocytosis during blood donation	WBC 206.7×10³/µL, BM: chronic-phase CML, variant t(6;9;22), BCR::ABL1 94.5%	Imatinib initiated	Hematologic response
Dec 2017	Insurance interruption	Lost access to imatinib	Treatment held ~1 month	Re-established care
Jan 2018	Hematology follow-up	Tolerating therapy	Restarted imatinib 400 mg daily	Continued response
Mar 2018	Follow-up	BCR::ABL1 58.8%	Continued imatinib	Stable chronic phase
Aug 2019	Lost follow-up	Stopped imatinib because of insurance	No treatment	Prolonged treatment interruption
Jul 2023	Hospitalization for hyperleukocytosis	WBC 443.5×10³/µL, BCR::ABL1 35.2%	Hydroxyurea, allopurinol; imatinib resumed	Cytoreduction achieved
Sept 2023	Right knee abscess	No abnormal myeloid cells on flow cytometry	Antibiotics	Infection resolved
Dec 2023	Neck cellulitis	CT neck with extensive soft tissue edema	IV antibiotics; temporary interruption of TKI	Restarted imatinib after discharge
May 2026	Current admission	Headache, intermittent fever, fatigue, abdominal distention, early satiety, massive splenomegaly, WBC 545×10³/µL	Hydroxyurea, TLS prophylaxis, bone marrow biopsy, flow cytometry	Persistent chronic-phase disease confirmed
May 2026	Definitive management	BM: MF-2 fibrosis, no blast transformation	Dasatinib planned/initiated	Outpatient hematology follow-up

Initial assessment and physical examination

Upon presentation, the patient reported several weeks of progressively worsening headaches accompanied by intermittent subjective fevers, abdominal distention, reduced appetite, fatigue, and early satiety. Initial vital signs demonstrated a low-grade fever (maximum temperature 38.0°C) and mild sinus tachycardia (heart rate approximately 105 beats/min), while blood pressure, respiratory rate, and oxygen saturation remained stable. He was alert, oriented, and in no acute distress.

Physical examination demonstrated tachycardia with a regular rhythm and normal respiratory effort without evidence of acute cardiopulmonary compromise. The abdomen was distended with marked splenomegaly but remained soft and non-tender without guarding or rebound tenderness. Bilateral thigh ecchymoses were noted without evidence of active bleeding. Neurologic examination was nonfocal, and no generalized lymphadenopathy was appreciated. Given the constellation of constitutional symptoms, massive splenomegaly, and persistent hematologic abnormalities, a comprehensive diagnostic evaluation was undertaken to assess disease status and exclude progression to advanced-phase CML.

Laboratory and diagnostic testing

Serial laboratory studies during hospitalization demonstrated persistent marked leukocytosis characterized by neutrophilia, basophilia, eosinophilia, monocytosis, and numerous immature granulocytic precursors. White blood cell counts remained markedly elevated, reaching approximately 342 ×10³/µL. Concurrent anemia was present, with hemoglobin measuring 7.4 g/dL and hematocrit 24.5%. Platelet counts remained preserved at approximately 398 ×10³/µL (Table 2).

**Table 2:** Laboratory findings at admission and during hospitalization Hematologic and biochemical laboratory findings during the patient's 2026 hospitalization for persistent chronic myeloid leukemia (CML). Admission values and ranges observed throughout hospitalization are shown. The patient presented with marked hyperleukocytosis, anemia, basophilia, eosinophilia, and elevated lactate dehydrogenase (LDH), consistent with active disease. Following initiation of therapy, white blood cell counts gradually decreased, while renal function and electrolyte levels remained stable. No laboratory evidence of clinically significant tumor lysis syndrome (TLS) was observed during hospitalization. Units are reported according to institutional laboratory reference standards. CML - chronic myeloid leukemia; LDH - lactate dehydrogenase; WBC - white blood cell count; ANC - absolute neutrophil count; BUN  - blood urea nitrogen; AST - aspartate aminotransferase; ALT - alanine transaminase; CRP - C-reactive protein

Parameter (unit)	Admission	Peak/nadir during hospitalization	Discharge	Reference range
WBC (×10³/µL)	342.0	545.2 (peak)	243	4.0-11.0
Hemoglobin (g/dL)	7.4	7.4–8.5	8.1	13.5-17.5
Platelets (×10³/µL)	261	585 (peak)	400	150-400
Blasts (%)	0–1	6 (peak)	2	<1
Basophils (%)	4	10 (peak)	8	<1
Eosinophils (%)	4	6 (peak)	4	0-5
Absolute basophils (×10³/µL)	13.68	21.81 (peak)	20.57	<0.20
LDH (U/L)	1,720	3,037 (peak)	645	140-280
Uric acid (mg/dL)	7.1	10.2 (peak)	4.3	3.5-7.2
AST (U/L)	37	180 (peak)	56	10-40
ALT (U/L)	20	180 (peak)	93	7-56
Creatinine (mg/dL)	0.90	1.00	0.90	0.7-1.3
CRP (mg/dL)	4.9	4.9	0.8	<1.0

Comprehensive infectious disease testing was negative, including HIV-1/2 antigen-antibody screening, HIV RNA testing, hepatitis B surface antigen, hepatitis B core IgM antibody, hepatitis C antibody, and hepatitis C viral RNA testing. Blood cultures showed no documented microbiologic growth (Table 3).

**Table 3:** Infectious disease screening results during hospitalization Comprehensive infectious workup, including testing for human immunodeficiency virus (HIV), hepatitis B virus (HBV), and hepatitis C virus (HCV), was negative. Blood cultures demonstrated no microbial growth. These findings excluded concurrent infectious etiologies and supported initiation of disease-directed hematologic therapy. HIV - human immunodeficiency virus; PCR - polymerase chain reaction; HBsAg - hepatitis B surface antigen; HBcAb - hepatitis B core antibody; HCV - hepatitis C virus

Test	Result	Interpretation
HIV-1/2 antigen/antibody screen	Nonreactive	Negative for HIV infection
HIV-1 RNA PCR	Not detected	No evidence of active HIV infection
Hepatitis B surface antigen (HBsAg)	Nonreactive	No evidence of active hepatitis B infection
Hepatitis B core IgM antibody (HBcAb IgM)	Nonreactive	No evidence of acute hepatitis B infection
Hepatitis C antibody (Anti-HCV)	Nonreactive	Negative hepatitis C screening
Hepatitis C virus RNA PCR	Not detected	No evidence of active hepatitis C infection
Blood cultures	No growth	No microbiologic evidence of bacteremia

Repeat bone marrow examination confirmed persistent/recurrent chronic myeloid leukemia without evidence of progression to accelerated-phase or blast-phase disease. Histopathologic evaluation demonstrated a markedly hypercellular marrow with an overall cellularity of approximately 95%, showing trilineage hematopoiesis with prominent myeloid hyperplasia and markedly decreased erythroid precursors. Myeloid elements exhibited complete maturation with left shift and no overt dysplasia. Megakaryocytes were scattered and included occasional small hypolobated forms. Peripheral blood smear examination demonstrated marked neutrophilia with left-shifted granulopoiesis and basophilia. Immunohistochemical staining identified rare CD34-positive blasts representing approximately 1% of marrow cellularity, while PAX5 and CD34 immunostains were otherwise negative. Collectively, these findings were consistent with persistent chronic-phase CML without morphologic evidence of leukemic transformation (Table 4).

**Table 4:** Bone marrow pathology findings during current hospitalization The marrow was markedly hypercellular (95%) with trilineage hematopoiesis, prominent myeloid hyperplasia, decreased erythropoiesis, and moderate reticulin fibrosis (MF-2). Rare CD34-positive blasts (~1%) were identified by immunohistochemistry without evidence of accelerated-phase or blast-phase transformation. Bone marrow aspirate differential analysis could not be performed because of inadequate aspirate material CML - chronic myeloid leukemia; IHC - immunohistochemistry; MF - myelofibrosis grade

Parameter	Finding	Reference/normal
Bone marrow diagnosis	Persistent/recurrent chronic myeloid leukemia without increase in blasts	N/A
Marrow cellularity	Approximately 95% (hypercellular)	Age-adjusted expected: ~30-70%
Hematopoiesis	Trilineage hematopoiesis present	Normal finding
Myeloid series	Marked myeloid hyperplasia with left-shifted maturation	No significant myeloid hyperplasia
Erythroid series	Markedly decreased	Normal trilineage representation
Megakaryocytes	Scattered, including occasional small hypolobated forms	Normal morphology
Lymphocytes	Small mature lymphocytes without atypical aggregates	Normal
CD34-positive blasts (IHC)	Approximately 1% of marrow cellularity	<5% consistent with chronic phase
PAX5	Negative	Negative
Reticulin fibrosis	MF-2 (moderate fibrosis)	MF-0
Ring sideroblasts	Not detected	Absent
Marrow dysplasia	No overt dysplasia identified	Absent
Blast transformation	Not identified	Absent
Bone marrow aspirate differential	Not performed due to inadequate aspirate smear	N/A
Pathologist interpretation	Persistent chronic-phase CML without increase in blasts	N/A

Additional marrow studies demonstrated moderate reticulin fibrosis (MF-2), indicative of ongoing chronic myeloproliferative activity. Bone marrow aspirate differential analysis could not be performed because of inadequate aspirate smears; however, concurrent peripheral blood studies revealed marked leukocytosis (235 ×10³/µL) with neutrophilia, immature myeloid forms (17%), eosinophilia (5%), and basophilia (6%). Correlation with cytogenetic and molecular studies was recommended for comprehensive assessment of persistent disease burden and therapeutic planning (Table 4). 

**Figure 1:**
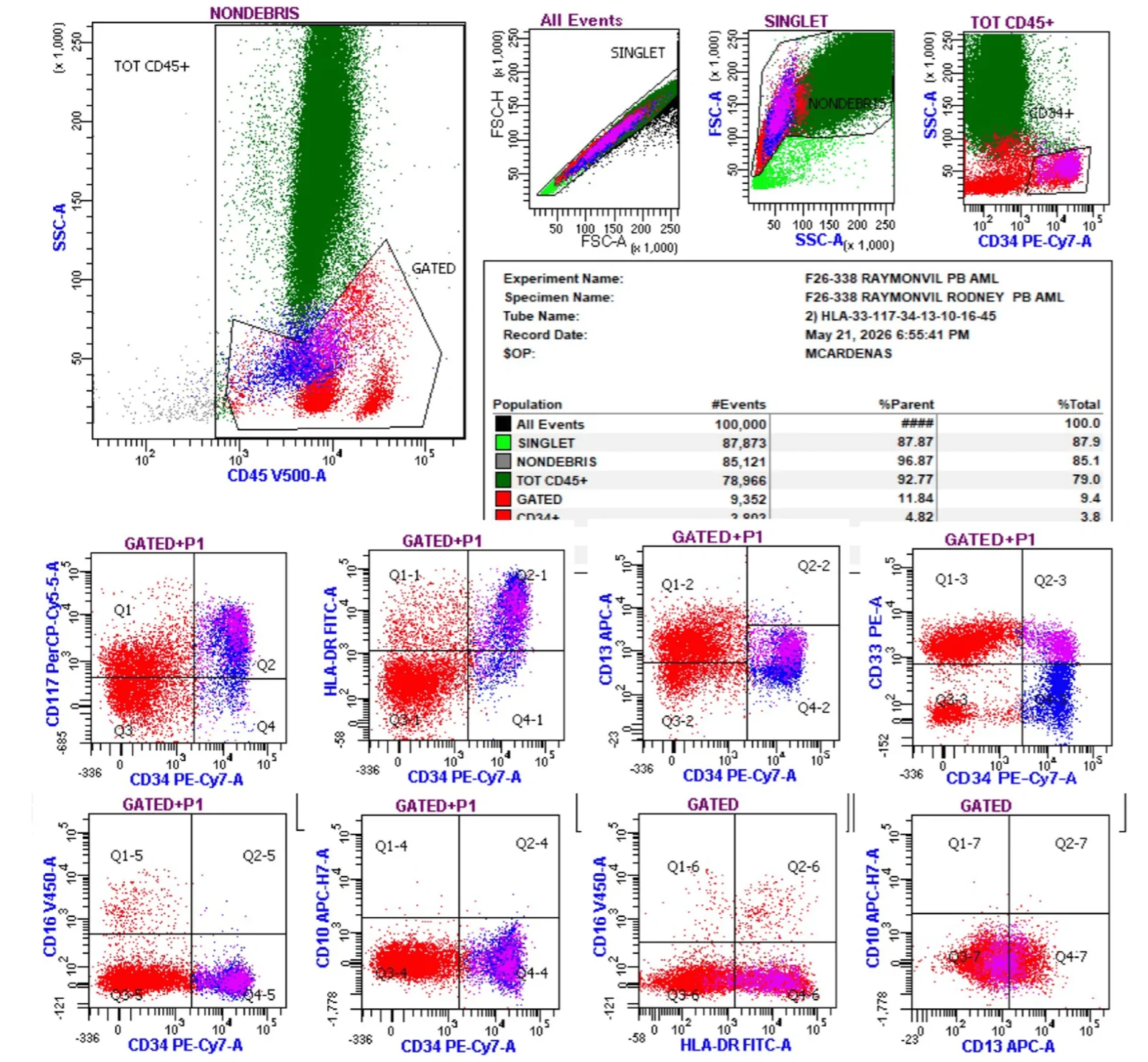
Flow cytometric immunophenotypic analysis of peripheral blood at hospital presentation Representative multiparameter flow cytometry of the peripheral blood obtained during the patient's 2026 hospitalization. Sequential gating was performed to exclude debris and doublets, followed by identification of the CD45-positive leukocyte population. Analysis demonstrated a small population of CD34-positive blasts (approximately 1%) expressing CD13, CD33, CD117, HLA-DR, and CD10, consistent with chronic-phase chronic myeloid leukemia (CML). The predominant cell population consisted of mature and maturing myeloid cells without immunophenotypic evidence of acute leukemic transformation.

**Figure 2:**
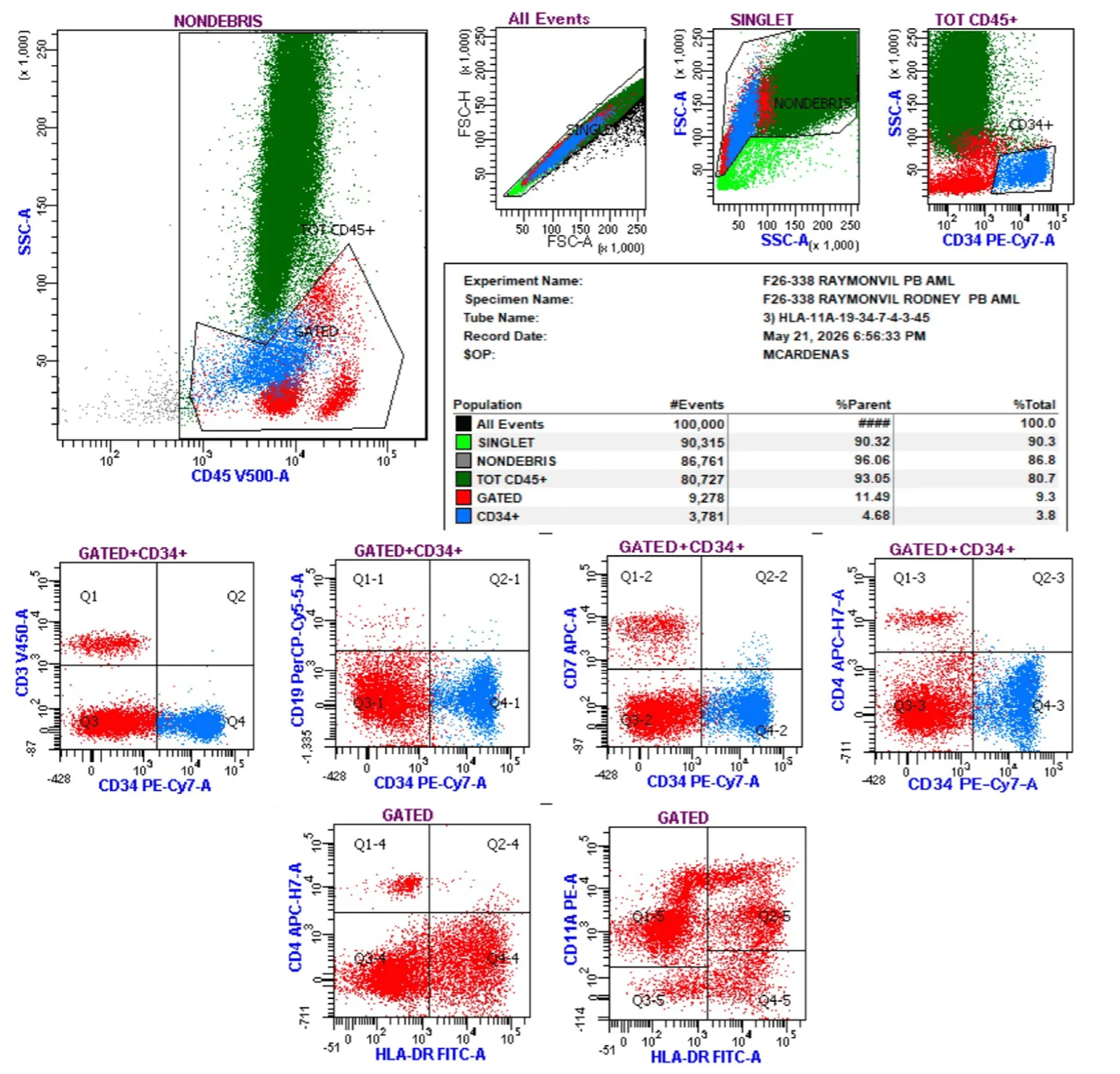
Immunophenotypic characterization of the CD34-positive blast population by multiparameter flow cytometry Representative multiparameter flow cytometric analysis of the gated CD34-positive blast population from peripheral blood obtained during the patient's 2026 hospitalization. Sequential gating identified the CD34-positive population, which accounted for approximately 4.7% of the gated events. The blast population demonstrated expression of CD34, HLA-DR, CD13, CD33, CD117, and CD10, while lacking aberrant expression of lymphoid markers including CD3, CD4, CD7, and CD19. This immunophenotypic profile is consistent with chronic-phase chronic myeloid leukemia and shows no evidence of immunophenotypic features suggestive of acute leukemic transformation.

Peripheral blood flow cytometry identified an abnormal population of CD34-positive/CD117-positive myeloid blasts comprising 3.16% of analyzed leukocytes. These cells demonstrated aberrant expression characterized by decreased CD33 and CD13 expression and were interpreted as abnormal myeloid blasts supporting the presence of an underlying myeloid neoplasm. No abnormal B-cell or T-cell populations were detected. The blast population showed an immature myeloid immunophenotype with expression of CD34, CD117, HLA-DR, CD13, and CD33, while lacking immunophenotypic evidence of lymphoid malignancy. Basophils were also increased, accounting for approximately 3.3% of analyzed events. Taken together with the bone marrow morphology, these findings supported persistent chronic-phase CML without evidence of accelerated-phase or blast-phase transformation (Figure 1 and 2; Table 5).

**Table 5:** Peripheral blood flow cytometric findings Flow cytometry identified an abnormal population of CD34-positive/CD117-positive immature myeloid blasts comprising 3.16% of analyzed leukocytes, with aberrantly decreased expression of CD33 and CD13. Basophils accounted for 3.3% of analyzed events. No abnormal B-cell or T-cell populations were detected. The immunophenotypic findings supported the presence of a persistent myeloid neoplasm and, when interpreted in conjunction with bone marrow morphology and cytogenetic studies, were consistent with persistent chronic-phase chronic myeloid leukemia without evidence of accelerated-phase or blast-phase transformation. AML - acute myeloid leukemia; CML - chronic myeloid leukemia; CD - cluster of differentiation; HLA-DR - human leukocyte antigen-DR; PB - peripheral blood

Parameter	Finding	Reference/interpretation
Specimen	Peripheral blood	-
Flow cytometry panel	Acute myeloid leukemia (AML) screen	-
Abnormal myeloid blasts	3.16% of analyzed leukocytes	Elevated; chronic-phase CML typically <10% blasts
Blast phenotype	CD34+, CD117+ immature myeloid blasts	Consistent with myeloid progenitors
CD33 expression	Decreased	Aberrant expression
CD13 expression	Decreased	Aberrant expression
Basophils	3.3% of analyzed events	Increased
Abnormal B-cell population	Not detected	Negative
Abnormal T-cell population	Not detected	Negative
Evidence of lymphoid malignancy	Not identified	Negative
Overall interpretation	Findings support persistent myeloid neoplasm	Consistent with persistent chronic-phase CML
Blast-phase transformation	Not identified	Blasts <10%
Accelerated-phase transformation	Not identified by flow cytometry	No immunophenotypic evidence

**Figure 3:**
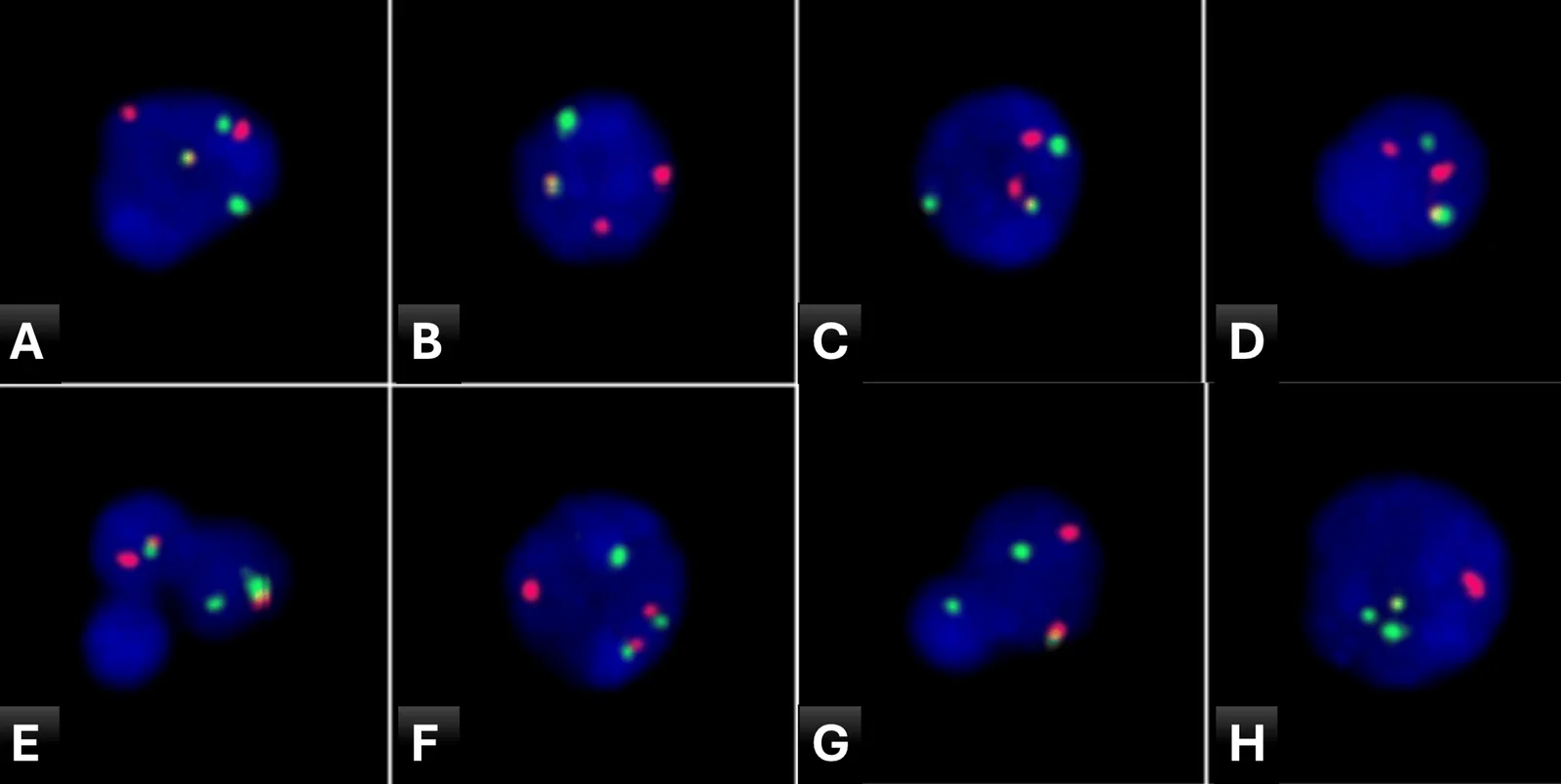
Fluorescence in situ hybridization (FISH) analysis demonstrating persistent BCR::ABL1-positive chronic myeloid leukemia Representative interphase nuclei stained with dual-color dual-fusion BCR::ABL1 probes are shown. Red signals represent ABL1, green signals represent BCR, and yellow signals represent fusion events. Panels A-H illustrate representative abnormal signal configurations observed during analysis. The dominant signal pattern was 1F2R2G, detected in 71.5% of analyzed nuclei, with additional variant fusion patterns identified in smaller subclonal populations. Overall, 91.0% of analyzed cells demonstrated abnormal BCR::ABL1 fusion signals, confirming persistence of the Philadelphia chromosome–positive clone despite maintenance of chronic-phase morphology.

Fluorescence in situ hybridization (FISH) analysis for BCR::ABL1 rearrangement was performed on 200 interphase nuclei and demonstrated persistent cytogenetic evidence of disease. The predominant signal pattern was 1F2R2G, identified in 143 of 200 cells (71.5%), with additional abnormal fusion signal patterns observed in smaller subclones. Overall, more than 70% of analyzed cells demonstrated evidence of BCR::ABL1 rearrangement, confirming persistence of the Philadelphia chromosome-positive leukemic clone despite prior therapy. These findings correlated with the morphologic and hematologic evidence of ongoing chronic-phase CML. Table 6 and Figure 3 summarize FISH analysis. 

**Table 6:** Results of repeat BCR::ABL1 FISH analysis Results of repeat fluorescence in situ hybridization (FISH) analysis using dual-color dual-fusion BCR::ABL1 probes. A total of 200 interphase nuclei were evaluated. The predominant signal configuration was the classical 1F2R2G fusion pattern, identified in 143 cells (71.5%). Overall, 182 of 200 analyzed nuclei (91.0%) demonstrated abnormal fusion patterns consistent with persistent BCR::ABL1 rearrangement, confirming a substantial residual Philadelphia chromosome-positive leukemic clone. F - fusion signal, R - ABL1 signal, G - BCR signal; FISH - fluorescence in situ hybridization

FISH signal pattern	Interpretation	Number of cells (n=200)	Percentage (%)
1F2R2G	Typical dual-fusion BCR::ABL1-positive pattern	143	71.5
1F2R1G	Variant BCR::ABL1-positive pattern	18	9.0
R1G2F1	Variant BCR::ABL1-positive pattern	16	8.0
2R2G	Normal signal pattern	9	4.5
2F1R1G	Variant BCR::ABL1-positive pattern	8	4.0
Other abnormal fusion patterns*	Variant BCR::ABL1-positive patterns	6	3.0
Total abnormal cells	Evidence of BCR::ABL1 rearrangement	182	91.0
Total normal cells	No detectable rearrangement	9	4.5
Unclassified/rare patterns	Miscellaneous signal configurations	9	4.5

**Figure 4:**
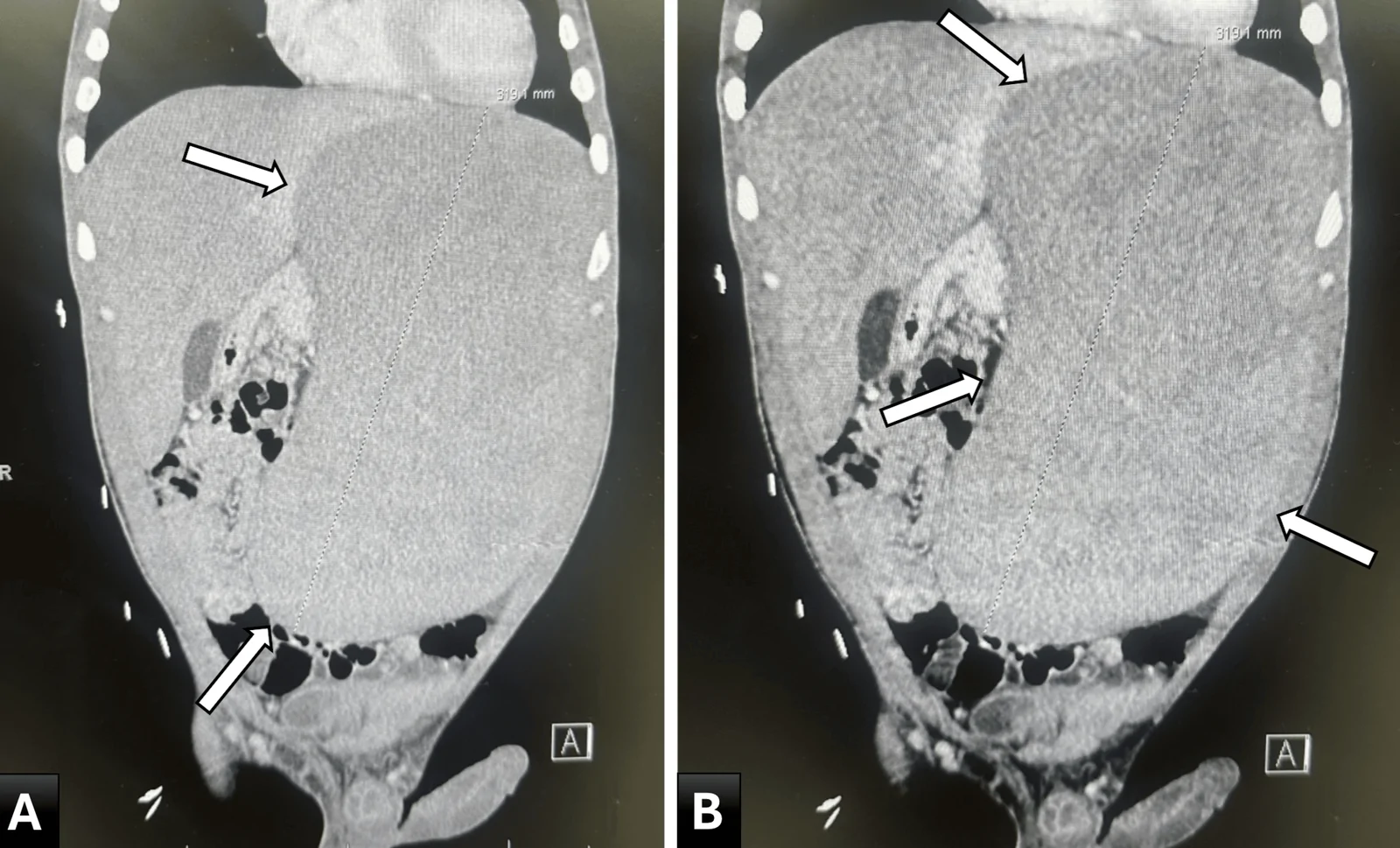
Massive splenomegaly on contrast-enhanced computed tomography in persistent chronic myeloid leukemia Coronal contrast-enhanced computed tomography (CT) images of the abdomen and pelvis demonstrating massive splenomegaly in a patient with persistent chronic myeloid leukemia. (A) Coronal CT image showing marked enlargement of the spleen extending from the left upper quadrant into the pelvis. (B) Coronal CT image at a different level further illustrating the extent of splenic enlargement and associated displacement of adjacent abdominal viscera. The spleen measured approximately 31.9 cm in maximal craniocaudal dimension. White arrows indicate the enlarged spleen and its mass effect on surrounding structures.

Additional diagnostic studies were performed to assess disease burden and evaluate associated symptoms. Contrast-enhanced computed tomography (CT) of the abdomen and pelvis demonstrated massive splenomegaly measuring approximately 31.9 cm in maximal craniocaudal dimension, occupying a substantial portion of the abdominal cavity and exerting significant mass effect on adjacent viscera (Figure 4). Borderline enlarged pelvic lymph nodes were also identified. The marked splenic enlargement was consistent with persistent myeloproliferative disease activity.

Magnetic resonance imaging (MRI) of the brain was obtained because of persistent headaches and was limited by artifact from dental braces but revealed no gross intracranial mass, midline shift, or other acute abnormality. 

Transthoracic echocardiography demonstrated preserved left ventricular systolic function with an estimated ejection fraction of 60-65%, mild right ventricular dilation, and no significant valvular abnormalities. 

Chest radiography showed no acute cardiopulmonary abnormalities, and electrocardiography demonstrated sinus tachycardia without other significant abnormalities.

Multidisciplinary board discussion

The case was reviewed in a multidisciplinary setting involving hematology, pathology, and laboratory medicine specialists. The principal diagnostic consideration was distinguishing persistent chronic-phase disease from progression to accelerated or blast phase. Despite substantial leukocytosis and persistent marrow involvement, multiple pathologic assessments demonstrated blast counts below established thresholds for disease acceleration. Bone marrow morphology, immunohistochemistry, and flow cytometry consistently identified only 1-2% blasts, favoring persistent chronic-phase CML rather than transformation.

The presence of moderate reticulin fibrosis and sustained hypercellularity raised concerns regarding disease persistence despite therapy. The board concluded that the overall findings were most consistent with recurrent or persistent chronic-phase CML with associated myelofibrotic change and recommended correlation with cytogenetic and molecular studies to further characterize disease status and guide subsequent treatment decisions.

Management

Given the patient's marked leukocytosis and substantial disease burden, cytoreductive therapy with hydroxyurea was initiated upon admission. He was closely monitored with serial complete blood counts and metabolic panels to assess treatment response and detect potential complications. Due to the high risk of tumor lysis syndrome associated with rapid cytoreduction, prophylactic measures including aggressive intravenous hydration and allopurinol were implemented, with frequent monitoring of serum uric acid, electrolytes, phosphorus, calcium, creatinine, and lactate dehydrogenase levels throughout hospitalization.

Following diagnostic reassessment and confirmation of persistent chronic-phase disease, treatment with dasatinib 100 mg daily was initiated as definitive disease-directed therapy. Antimicrobial prophylaxis with acyclovir was also administered during treatment. Supportive care included ongoing monitoring of hematologic parameters, renal function, and treatment-related adverse effects. The patient remained under the care of a multidisciplinary hematology team, with therapeutic decisions guided by serial laboratory assessment and clinical response.

Outcome and follow-up 

The patient demonstrated a favorable hematologic response to treatment during hospitalization. Following initiation of hydroxyurea and subsequent dasatinib therapy, his leukocyte count progressively declined from greater than 500 ×10³/µL at presentation to approximately 243 ×10³/µL by discharge. Concurrently, his presenting symptoms, including headache and abdominal discomfort, improved. Despite the substantial disease burden and rapid cytoreduction, no clinically significant tumor lysis syndrome developed, and renal function remained stable throughout the hospital course.

After stabilization, the patient was discharged on dasatinib 100 mg daily with close outpatient hematology follow-up. Ongoing surveillance was recommended, including serial complete blood counts, comprehensive metabolic panels, lactate dehydrogenase, uric acid, and quantitative BCR::ABL1 transcript monitoring by real-time PCR to assess molecular response, treatment efficacy, and disease control over time. Although longitudinal BCR::ABL1 transcript measurements were not available during the present hospitalization, serial molecular monitoring was recommended for subsequent follow-up in accordance with current CML management guidelines. Continued adherence to tyrosine kinase inhibitor therapy and longitudinal hematologic follow-up were emphasized to reduce the risk of disease progression and optimize long-term outcomes. 

## Discussion

Background (history, epidemiology, and risk factors)

Chronic myeloid leukemia (CML) is an uncommon myeloproliferative neoplasm defined by the Philadelphia chromosome and the resulting BCR::ABL1 fusion gene [1-3]. In the United States, the overall age-adjusted incidence of CML is approximately 2.1 cases per 100,000 persons per year, and CML accounts for a small fraction of all new cancer diagnoses [1-3]. The disease is strongly age-associated: population-based data show that CML is most frequently diagnosed between 65 and 74 years of age, with a median age at diagnosis of approximately 67 years [1-3]. By contrast, diagnosis in younger adults is uncommon; SEER data indicate that patients aged 20-34 years account for only 6.7% of new CML cases, while those younger than 20 years account for 1.6% [4,6,7].

CML in adolescents and young adults is increasingly recognized as a clinically important subgroup because younger patients may present with distinct disease features [4,6,7]. Several studies have reported that younger patients can present with higher leukocyte counts, larger spleen size, and greater disease burden at diagnosis compared with older adults [4,6,7]. Despite this, outcomes have improved substantially in the tyrosine kinase inhibitor era, and long-term survival can approach that of the general population when optimal molecular responses are achieved [1,3,4,6,7].

Few established risk factors for CML have been identified. Ionizing radiation remains the most consistently recognized environmental risk factor, whereas hereditary predisposition and lifestyle-related exposures have not been definitively linked to disease development [1,2]. Because CML is rarely diagnosed in young adults, cases with prolonged disease course, persistent high disease burden, and unusual cytogenetic features provide important insight into the clinical heterogeneity and long-term management challenges of CML in this population.

Pathology/pathophysiology

Chronic myeloid leukemia (CML) is a clonal myeloproliferative neoplasm driven by the BCR::ABL1 fusion gene, most commonly generated by the Philadelphia chromosome translocation t(9;22)(q34;q11.2) [1-3]. Constitutive BCR::ABL1 tyrosine kinase activity activates multiple downstream signaling pathways, promoting uncontrolled myeloid proliferation and producing the characteristic features of CML, including leukocytosis, left-shifted granulopoiesis, basophilia, and splenomegaly [1,3,15].

Variant Philadelphia chromosome translocations occur in approximately 5-10% of patients and involve one or more chromosomes in addition to chromosomes 9 and 22 while preserving formation of the BCR::ABL1 fusion gene [8-10]. Although most patients respond similarly to tyrosine kinase inhibitor therapy, the biologic significance of many rare variant rearrangements remains incompletely understood [8-10].

In this case, a rare three-way translocation, t(6;9;22)(p21;q34;q11.2), was associated with persistent BCR::ABL1-positive chronic-phase CML. Marked leukocytosis, basophilia, massive splenomegaly, and a hypercellular marrow reflected ongoing myeloproliferative activity, while low blast counts on bone marrow examination and flow cytometry confirmed persistent chronic-phase disease without transformation to accelerated- or blast-phase CML [1,3].

Comparative analysis of this case with the existing literature

Clinical Presentation

The patient's initial presentation with incidental leukocytosis detected in 2017 during a routine blood donation screening is consistent with reports indicating that many patients with CML are 50% asymptomatic at diagnosis [5]. However, the degree of leukocytosis observed in this case being 187.8 ×10³/µL at diagnosis and >500 ×10³/µL during recurrence, and the presence of massive splenomegaly measuring 31.9cm demonstrates a high disease burden. CML most commonly presents in middle-aged and older adults, with a median age of onset around 60-65 [5]. Initial diagnosis of our patient was when he was 24, placing him within a small subset of cases.

CML found in individuals under 30 is extremely rare, with an incidence estimated at approximately 0.025 cases per 100,000 [5]. Identification of a complex variant Ph (vPh) chromosome, t(6;9;22)(q34;q11.2;p21), distinguishes our patient's case from most CML cases having the traditional t(9;22) translocation. Only 5-8% of CML cases have complex variant translocations, and chromosome 6 involvement is particularly uncommon [5,12-14]. Strikingly, the patient presented with a CML diagnosis at 24 with a vPH chromosome t(6;9;22) estimated to be approximately 0.025-0.08 cases per 100,000 population per year, noting the rarity of this presentation. Additionally, despite the nine-year disease course this patient had along with remarkable hematologic levels, the patient had no evidence of accelerated-phase or blast-phase transformation considering the uncommon nature of this case. 

Diagnosis

The diagnostic evaluation in our patient was consistent with routine diagnostic workup for CML. This included complete blood count, peripheral blood smear, bone marrow biopsy, flow cytometry, cytogenetics, and Fluorescence In Situ Hybridization (FISH) analysis. Bone marrow examination demonstrated hypercellularity with granulocytic hyperplasia. Cytogenetic analysis identified vPh chromosome translocation, t(6;9;22)(q34;q11.2;p21). Additionally, FISH confirmed BCR-ABL fusion positivity in 94.5% of the analyzed cells. Comparable studies of CML and vPh chromosome CML have noted cytogenetic and FISH analysis confirmation along with molecular confirmation [5,12,13]. Re-evaluation in 2026 consisted of acknowledging that the chronic-phase of CML was maintained without blast transformation, demonstrating moderate reticulin fibrosis (MF-2) and CD34-positive blasts approximately 1% on immunohistochemistry. 

In addition to standard protocol testing, advanced imaging was utilized to evaluate disease burden in our patient. Contrast-enhanced CT imaging of the abdomen displayed massive splenomegaly measuring approximately 31.9cm with significant mass effect.

Splenomegaly is a common manifestation of CML and reflects extramedullary hematopoiesis and leukemic cell sequestration [5,6,13]. However, the degree of enlargement noted in this patient is more pronounced than that described in most reported cases, reflecting persistent disease activity.

*Management* 

Young adults with CML often present with a higher disease burden than older patients, including greater leukocytosis and splenomegaly. In recent studies, tyrosine-kinase inhibitors (TKI) therapy has improved the prognosis of all age groups diagnosed with CML. Our patient was initiated with dasatinib 100 mg daily during his 2026 re-evaluation. Other documented cases of young adults with CML and/or vPH chromosome t(6;9;22) have been treated with first-generation TKIs, such as Imatinib [6,7]. Compared with Imatinib, Dasatinib is more potent and is commonly utilized for its ability to induce faster and deeper molecular responses for chronic-phase CML. Furthermore, hydroxyurea was initiated prior to TKI therapy for rapid cytoreduction in the setting of extreme hyperleukocytosis, as seen in our patient, prior to definitive therapy with dasatinib. 

Current studies have shown no significant difference in survival rates between young adults and older patients diagnosed with CML [6,7]. Early initiation and compliance with TKIs aid in long-term disease control and improved quality of life for patients diagnosed under the age of 30 with higher leukocyte counts and more pronounced organomegaly [5,7]. In this case, the patient presented at a young age for initial diagnosis, with marked leukocytosis, massive splenomegaly measuring approximately 31.9 cm, despite high disease burden. Notably, despite a nine-year disease course, marked leukocytosis, massive splenomegaly, persistent BCR::ABL1 positivity, and moderate marrow fibrosis, the patient remained in chronic phase without evidence of accelerated-phase or blast-phase transformation. 

*Outcome and Follow-up* 

The patient demonstrated significant clinical and hematologic improvement following cytoreduction and initiation of dasatinib therapy, with a marked decline in leukocyte count and resolution of his presenting symptoms. He was discharged with plans for close hematology follow-up, including serial complete blood count monitoring, comprehensive metabolic panels, lactate dehydrogenase, uric acid, and serial quantitative BCR::ABL1 transcript monitoring by real-time polymerase chain reaction (RT-qPCR) using the International Scale (IS) to evaluate treatment response and long-term disease control. Although longitudinal quantitative BCR::ABL1 transcript results were unavailable for inclusion in this report, continued molecular monitoring was recommended to determine treatment response and achievement of standardized molecular milestones, including major molecular response (MMR; BCR::ABL1 ≤0.1% IS), MR4 (≤0.01% IS), and MR4.5 (≤0.0032% IS), during long-term follow-up.

This case highlights several features described in the current literature on chronic myeloid leukemia (CML) in adolescents and young adults (AYA). Although CML is predominantly diagnosed in older adults, younger patients frequently present with higher leukocyte counts, more pronounced splenomegaly, and greater disease burden at diagnosis [4,6,7]. Consistent with these observations, our patient was diagnosed at 24 years of age and subsequently presented with extreme leukocytosis and massive splenomegaly. Notably, despite persistent disease nearly nine years after diagnosis and the presence of a rare variant Philadelphia chromosome, t(6;9;22), repeat bone marrow examination, flow cytometry, and fluorescence in situ hybridization (FISH) consistently demonstrated persistent chronic-phase disease without evidence of accelerated- or blast-phase transformation. Similar variant Philadelphia chromosome translocations have been reported only rarely, and their long-term prognostic significance remains incompletely understood [8-10,12,13].

*Summary* 

This case highlights several uncommon features of chronic myeloid leukemia, including diagnosis at 24 years of age, the presence of a rare three-way variant Philadelphia chromosome translocation t(6;9;22)(p21;q34;q11.2), and persistence of chronic-phase disease nearly nine years after diagnosis. Consistent with reports describing adolescents and young adults with CML, the patient exhibited a substantial disease burden characterized by marked leukocytosis and massive splenomegaly. However, unlike many cases in which such findings raise concern for disease progression, repeat bone marrow evaluation, peripheral blood flow cytometry, and cytogenetic studies demonstrated persistent chronic-phase disease without evidence of accelerated-phase or blast-phase transformation. Comparison with the existing literature suggests that although variant Philadelphia chromosome translocations are uncommon and their long-term clinical significance remains incompletely understood, durable chronic-phase disease can be maintained despite significant hematologic and morphologic abnormalities. This case underscores the importance of comprehensive molecular surveillance and long-term follow-up in young patients with CML. Although longitudinal quantitative BCR::ABL1 transcript data were unavailable in this case, repeat FISH, bone marrow morphology, and flow cytometric findings consistently demonstrated persistent chronic-phase disease without evidence of accelerated- or blast-phase transformation. Table 7 is a summary of the comparison between this case and the current literature.

**Table 7:** Comparison of the present case with findings reported in the literature CML - chronic myeloid leukemia; FISH - fluorescence in situ hybridization; MF - myelofibrosis grade; TKI - tyrosine kinase inhibitor; WBC - white blood cell count

Domain	Findings reported in the literature	Present case
Age at diagnosis	Median age at diagnosis is approximately 60–65 years; patients younger than 30 years represent a small minority of cases [1-7].	Diagnosed at 24 years of age.
Initial presentation	Approximately 40–50% of patients are asymptomatic and diagnosed incidentally through routine laboratory testing [1-3].	Leukocytosis was incidentally detected during blood donation.
Leukocyte count	Leukocytosis is common; younger patients often present with higher leukocyte counts than older adults [4,6,7].	WBC 187.8 ×10³/µL at diagnosis and >500 ×10³/µL during hospitalization.
Splenomegaly	Common manifestation of CML; often more pronounced in younger patients [4,6,7].	Massive splenomegaly measuring 31.9 cm with mass effect on adjacent organs.
Cytogenetics	Classic Philadelphia chromosome t(9;22)(q34;q11.2) is present in >90% of cases. Variant translocations occur in approximately 5-10% of patients [1,8–11].	Rare three-way variant Philadelphia chromosome t(6;9;22)(p21;q34;q11.2).
Chromosome 6 involvement	Reported only in isolated case reports and small case series [10,12,13].	Presence of rare t(6;9;22) translocation.
Bone marrow findings	Hypercellular marrow with granulocytic hyperplasia and left-shifted myelopoiesis [1-3].	Markedly hypercellular marrow (95%) with myeloid predominance and MF-2 fibrosis.
Disease phase	Most patients present in chronic phase; progression to accelerated or blast phase may occur over time. [1–3]	Persistent chronic-phase disease despite a 9-year disease course.
Molecular findings	BCR::ABL1 positivity confirmed by FISH and/or molecular testing [1-3].	BCR::ABL1 positivity confirmed at diagnosis and persisted on repeat FISH testing.
Treatment	TKI therapy is the standard of care; younger patients generally achieve outcomes comparable to older adults when adequately treated [1-3,6,7].	Treated with hydroxyurea for cytoreduction followed by dasatinib 100 mg daily.
Outcome	Long-term survival is favorable with sustained molecular response [1-3].	Clinical and hematologic improvement achieved; persistent chronic-phase disease without accelerated- or blast-phase transformation after nearly 9 years.
Unique feature	Rare reports describe variant Philadelphia chromosome translocations involving chromosome 6 [10,12,13].	Combination of young age at diagnosis (24 years), rare t(6;9;22) translocation, massive splenomegaly, and persistent chronic-phase disease nearly 9 years after diagnosis.

What we learned from this case 

This case illustrates several important lessons regarding the long-term management of chronic myeloid leukemia (CML) in young adults. First, although CML is predominantly diagnosed in older individuals, it can occur in patients in their twenties and may initially be detected incidentally. The diagnosis of CML in a 24-year-old patient highlights the importance of considering myeloproliferative neoplasms in the differential diagnosis of marked leukocytosis regardless of age.

Second, comprehensive cytogenetic and molecular characterization remains essential throughout the disease course. The identification of a rare three-way variant Philadelphia chromosome translocation, t(6;9;22)(p21;q34;q11.2), emphasizes the value of detailed cytogenetic analysis in defining uncommon disease subsets and guiding long-term surveillance.

Most importantly, this case demonstrates that a substantial disease burden does not necessarily indicate disease progression. Despite extreme leukocytosis, massive splenomegaly, persistent BCR::ABL1 positivity, and moderate marrow fibrosis, the patient remained in chronic phase nearly nine years after diagnosis without evidence of accelerated-phase or blast-phase transformation. This observation underscores the biologic heterogeneity of CML and reinforces the importance of disease-phase assessment using morphologic, immunophenotypic, cytogenetic, and molecular findings rather than relying solely on disease burden. Finally, continued molecular monitoring and adherence to tyrosine kinase inhibitor therapy remain critical for achieving durable long-term disease control in young patients with persistent CML.

Limitations

This report has several limitations inherent to retrospective single-patient case reports. Longitudinal quantitative BCR::ABL1 transcript measurements by real-time polymerase chain reaction (RT-qPCR) using the International Scale (IS) were unavailable for review, precluding documentation of standardized molecular response milestones such as major molecular response (MMR), MR4, and MR4.5 over the patient's nine-year disease course. Additionally, complete outpatient treatment records, including detailed timelines of tyrosine kinase inhibitor adherence, treatment interruptions, and serial molecular monitoring, were not consistently available. Nevertheless, the diagnosis and longitudinal assessment of disease persistence were supported by concordant clinical findings, serial hematologic parameters, repeat bone marrow morphology, peripheral blood flow cytometry, cytogenetic analysis, fluorescence in situ hybridization (FISH), and cross-sectional imaging. Despite these limitations, this case provides valuable long-term clinical insight into a young patient with chronic-phase CML harboring a rare three-way variant Philadelphia chromosome translocation, t(6;9;22)(p21;q34;q11.2).

## Conclusions

This case describes a young adult diagnosed with BCR::ABL1-positive chronic-phase chronic myeloid leukemia at 24 years of age harboring a rare three-way variant Philadelphia chromosome translocation, t(6;9;22)(p21;q34;q11.2). Despite a nine-year disease course characterized by marked leukocytosis, massive splenomegaly, persistent BCR::ABL1 positivity, and moderate marrow fibrosis, repeat evaluation demonstrated persistent chronic-phase disease without evidence of accelerated-phase or blast-phase transformation. This case highlights the clinical heterogeneity of CML and illustrates that significant disease burden does not necessarily correlate with disease progression. Furthermore, it contributes to the limited literature describing chromosome 6-associated variant Philadelphia chromosome translocations and their long-term clinical behavior. Continued molecular monitoring, cytogenetic assessment, and adherence to tyrosine kinase inhibitor therapy remain essential for optimizing long-term outcomes in young patients with persistent CML.

## References

[1] Senapati J, Sasaki K, Issa GC, Lipton JH, Radich JP, Jabbour E, Kantarjian HM (2023). Management of chronic myeloid leukemia in 2023 - common ground and common sense. Blood Cancer J.

[2] Hochhaus A, Saussele S, Rosti G (2018). Chronic myeloid leukaemia: ESMO Clinical Practice Guidelines for diagnosis, treatment and follow-up. Ann Oncol.

[3] Jabbour E, Kantarjian H (2024). Chronic myeloid leukemia: 2025 update on diagnosis, therapy, and monitoring. Am J Hematol.

[4] Abdulla MA, Aldapt MB, Chandra P (2024). Chronic myeloid leukemia in adolescents and young adults: clinicopathological variables and outcomes. Oncology.

[5] Patel K, Syed J, Jagadish A, Vedantam V, Daugherty D (2025). Chronic myeloid leukemia in a young adult. Cureus.

[6] Al-Rabi K, Ma'koseh M, Al-Qadi F (2024). Clinical characteristics and outcomes of CML in adolescents and young adults. Clin Lymphoma Myeloma Leuk.

[7] Nishiyama-Fujita Y, Nakazato T, Iriyama N (2022). Outcomes of adolescents and young adults with chronic-phase chronic myeloid leukaemia treated with tyrosine kinase inhibitors. Ann Med.

[8] Bahashwan SM (2024). Chronic myeloid leukemia with a rare Philadelphia chromosome variant involving chromosome 16. Am J Case Rep.

[9] Son HJ, Lee JH (2024). Novel four-way variant translocation, t(1;9;22;16)(q21;q34;q11.2;q24), in a patient with chronic myeloid leukemia. Diagnostics (Basel).

[10] Chen L, Zhang J, Yang N (2022). A unique three-way variant Philadelphia chromosome t(6;9;22)(p21.3;q34;q11.2) in a newly diagnosed patient with chronic myeloid leukemia responded to flumatinib. Onco Targets Ther.

[11] Dutta B, Khuraijam M (2024). Variant Philadelphia chromosome in nine cases of chronic myeloid leukemia: experience of a tertiary care hospital in eastern India. J Med Sci.

[12] Asif M, Hussain A, Wali A (2017). A rare case of three-way complex variant translocation in chronic myeloid leukemia t(6;9;22)(p21;q34;q11): a case report. Biomed Rep.

[13] Ciftciler R, Saglam EA, Inanc A, Ozcebe O, Haznedaroglu IC (2019). A unique case of complex variant translocation of t(6;9;22)(p22;q34;q11.2), der(19) in a newly diagnosed patient with chronic myeloid leukemia. Cancer Genet.

[14] Bhardwaj M, Mishra SK, Gupta A, Mehta P, Sharma S, Mohanty SK (2024). Three-way Philadelphia translocation [t(46, XX, t(9;22;16) (q34;q11.2;q24)] in chronic myeloid leukemia: a report of two cases with review of the literature. J Cancer Res Ther.

[15] Soverini S (2023). Resistance mutations in CML and how we approach them. Hematology Am Soc Hematol Educ Program.

